# Clinical relevance of brain MRI changes in primary central nervous system lymphoma after high-dose-chemotherapy and autologous stem cell transplantation

**DOI:** 10.1038/s41409-024-02382-4

**Published:** 2024-08-09

**Authors:** Sina A. Beer, Robert Möhle, Ghazaleh Tabatabai, David A. Merle, Ulrike Ernemann, Vivien Richter, Claudia Lengerke

**Affiliations:** 1 Department of Internal Medicine II, Hematology, Oncology, Clinical Immunology and Rheumatology, University Hospital Tübingen, Tübingen, Germany; 2 Department of Neurology and Interdisciplinary Neuro-Oncology, University Hospital Tübingen, Hertie Institute for Clinical Brain Research, Tübingen, Germany; 3 Center for Neuro-Oncology, Comprehensive Cancer Center Tübingen-Stuttgart, Tübingen, Germany; 4 Department of Ophthalmology, University Hospital Tübingen, Tübingen, Germany; 5 Department of Neuroradiology, University Hospital Tübingen, Tübingen, Germany

**Keywords:** Stem-cell research, B-cell lymphoma, Medical imaging, Quality of life

## Abstract

Primary central nervous system lymphoma (PCNSL) is a potentially curable disease, but affected patients often struggle in everyday life due to disease- and therapy-associated sequelae. High-dose chemotherapy followed by autologous stem cell transplantation (HDC/ASCT) is the standard consolidation therapy, replacing whole brain radiation therapy (WBRT) amongst others due to less long-term cognitive decline. Nevertheless, white matter lesions (WML) are common findings in brain MRI after HDC/ASCT, but their clinical significance remains underexplored. Here, we correlate WML and brain atrophy with neuropsychological and quality-of-life evaluations collected post-treatment. We found that a significant part of PNCSL long-term survivors develop a high WML burden after HDC/ASCT, but we fail to associate them with specific patient or therapy characteristics. Intriguingly, even a high WML burden does not seem to affect QoL, basic neurocognition testing or performance status negatively. These results contrast findings in previous neuroimaging studies on healthy and cancer patients.

## Introduction

The introduction of high-dose chemotherapy followed by autologous stem cell transplantation (HDC/ASCT) as first-line consolidation treatment has significantly improved overall survival in patients with primary central nervous system lymphoma (PCNSL) [1], leading to increased interest in their quality of life (QoL) after this treatment. Today, HDC/ASCT is preferred to early whole brain radiation therapy (WBRT) due to less treatment-related delayed neurotoxicity [2, 3]. However, drugs used in HDC regimens, such as methotrexate (MTX), may still have neurotoxic effects [4, 5]. Therefore, understanding the neurocognitive safety of HDC/ASCT in long-term survivors is critical [6]. Approximately half of all HDC/ASCT recipients experience acute neurocognitive deficits, irrespective of their specific diagnoses [7], with outcomes ranging from stability and recovery [8] to potential impairment [9–11]. For patients with PCNSL, Ferreri et al. demonstrated a positive long-term effect of ASCT on cognitive function and QoL [1], but 20-40% of HDC/ASCT recipients in this cohort exhibit cognitive impairment during long-term follow-up [7, 12]. A cumulative neurotoxic process, influenced by individual cognitive reserves such as older age, likely determines the severity of cognitive impairment [13, 14].

The preferred diagnostic modality for PCNSL is cranial MRI (cMRI) due to its sensitivity and high spatial resolution, which surpasses that of PET-CT used in other lymphoma subtypes [15]. cMRIs of PCNSL patients during follow-up frequently show white matter (WM) changes extending beyond scar tissue [7, 16], commonly in the supratentorial periventricular and deep WM [17]. These WM lesions (WML) are also observed in normal brain aging, with more than 90% of healthy adults over 80 years (y) showing WML, consistently associated with global cognitive decline [18–21]. Data about the association between WML and neurocognition in PCNSL patients after HDC/ASCT is heterogeneous [7, 10, 11, 22–25].

In sum, the etiology, evolution, and clinical significance of WML in PCNSL patients after HDC/ASCT remain largely unknown. Our study addresses this knowledge gap by analyzing long-term outcomes in PCNSL patients following HDC/ASCT through longitudinal cMRI, combined with QoL and neurocognitive evaluations of this common therapeutic regimen.

## Patients and methods

We performed a cross-sectional assessment of QoL and neurocognition, alongside a retrospective review of cMRI imaging data, in PCNSL-patients treated with HDC/ASCT at University Hospital Tübingen between 2006 and 2021.

### Imaging analysis

**Fig. 1:**
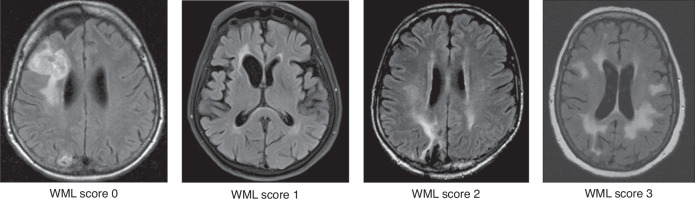
Exemplary MRI scans scored by the modified Fazekas score (mFS) from score 0 to 3 for the overall white matter lesion (WML) burden. All exemplary scans are from PCNSL patients. The assessment did not include specific lesions, such as tumor infiltration, post-therapeutic, or post-ischemic gliosis with surrounding edema.

cMRIs acquired at the following time points were reviewed: shortly before (baseline) and three months after receiving HDC/ASCT (post-HDC, maximum delay 14 days); at 12, 24, 36, 48, and 60 months (m12–m60) after the treatment, and for long-term survivors the last available imaging (>m60). After the review of the complete imaging protocol, T2-FLAIR images were rated visually for the presence of WML by a neuroradiologist (VR, 10 years of experience in neuroimaging) who was blinded to clinical information. A WML score at each imaging time point was defined using the Fazekas score for deep WM (FS-DWM, range 0–3) and periventricular WM (FS-PWM, range 0–3) as well as the modified Fazekas score (mFS, range 0–3) for the summarized WML burden (average of maximum of FS-PWM and FS-DWM, Fig. 1) [26]. WML assessment did not include lesions such as tumor infiltration, edema or post-therapeutic/-ischemic gliosis. Patients were assigned to a low (mFS 0–1) or a high WML burden (mFS 2–3) group. We calculated the maximum mFS change over time and documented the time point of any mFS conversion from low to high. Additionally, global cortical atrophy (GCA, range 0–3) and mesial temporal atrophy (MTA, range 0–4) were visually assessed at each time point [27]. For the DWM, PWM, mFS, GCA, the pathological threshold score was ≥2, for MTA, the threshold was ≥2 in patients younger than 75 y and ≥3 in those older than 75 y.

### Clinical evaluation

Previously defined cMRI responses were reevaluated utilizing the International PCNSL Collaborative Group (IPCG) Consensus Guidelines and rated as complete response (CR), unconfirmed CR (CRu), partial response (PR), stable disease (SD) or progress (PD) [28]. The bedside neurocognitive and QoL evaluation included an ECOG performance status ranging from 0 (with no restrictions) to 5 (death), a mini mental state examination (MMSE) ranging from 0 (worst performance) to 30 points (best performance), and patient-reported outcome (PRO) measurements (PROMs). ECOG scores were assessed at baseline and latest follow-up, while MMSE and PROMs were collected only at the latest follow-up. For PRO assessment the EORTC-QLQ30 (version 3) was used, with items 29 and 30 specifically measuring the global health status (GHS) of the patients. For detailed information on PROMs, we refer to the precursor project [29]. Electronic medical records were reviewed for applied chemotherapy drugs, MTX dosage, Charlson Comorbidity Index (CCI) and HCT-specific comorbidity index (HCT-CI) calculation [30, 31] as well as remission status using the following databases: The Comprehensive Cancer Center Tübingen (CCC), the Koordobas-System and the German Register for Stem Cell Transplantation (DRST). Patients who progressed in the meantime were excluded from the study. Patients who had a relapse before HDC/ASCT (*n* = 4) were included. The local Institutional Review Board approved this study (no. 376/2022BO2) and informed consent was obtained from all subjects.

### Statistics

Statistical analyses were performed using Excel (Microsoft Office Professional Plus v. 2312) and IBM® SPSS Statistics 29.0. Patient characteristics were expressed as frequencies or categorical variables. Categorical data was compared via Chi-Quadrat-test (χ^2^) or exact Fisher-test. Continuous variables are statistically examined using independent t-tests or ANOVA analysis. The WML burden impact on neurocognition and QoL was analyzed using Mann–Whitney-U-testing. We used multivariate analysis to determine the influence of various variables on the prevalence of WML. Associations were assessed by Spearman correlation. To evaluate the differences between paired observations, sign tests were utilized. Due to the limited sample size, non-parametric tests were used in most instances. All significance tests were two-sided, and a *p* value < 0.05 was considered significant.

## Results

### Patient characteristics and therapy regimes

Out of 40 eligible patients identified, 12 were excluded due to a lack of imaging acquired more than 12 months after HDC/ASCT. The remaining 28 patients were included in the analysis, with an average follow-up of 4.7 years (range 1–15 years) and an average age at HDC/ASCT of 57.1 y (25–77, including 3 patients >70 y). Detailed demographic and clinical information, as well as data on treatment regimens, are presented in Table 1. For Thiotepa-based conditioning, patients were classified into two groups: high-dose TT (4 × 5 mg/kg, *n* = 20) and low-dose TT (2 × 5 mg/kg, *n* = 8). None of our patients received an intrathecal therapy at any time before or after the ASCT.

**Table 1:** Demographic, clinical and treatment information.

**Demographic data**	
Patients included	*n* = 28
Gender, Female	42.3% (12/28)
Age at diagnosis, years - average (range), quartiles	60.5 (25–77), Q1 = 46.0, Q2 = 60.5, Q3 = 65.7
**Clinical data**	
Follow-up, years - average (range)	4.7 (1–15)
Histologic subtype, DLBCL	100% (28/28)
Deep brain structures involved	50% (14/28)
Baseline ECOG, grade - median (range)	1 (0–3)
- Subgroup: 0 or 1	82.1% (23/28)
Baseline CCI, points - median (range), subgroups	4 (2–8)
- Subgroup: 2 points	28.6% (8/28)
- Subgroup: 4 points	25% (7/28)
Baseline HCT-CI, points - median (range), subgroup	1 (0–5)
- Subgroup: high risk ( ≥ 3 points)	28.6% (8/28)
Average follow-up, years (range)	4.7 (1–15)
**Treatment data**	
HDC/ASCT, 1st line therapy	85.7% (24/28)
Induction treatment, Rituximab/MTX-containing	100% (28/28)
- MTX reduction	7.1% (2/28)
Consolidation treatment, TT-containing	100% (28/28)
- 4x5mg/kg (high dose)	71.4% (20/28)
- 2x5mg/kg (low dose)	28.6% (8/28)
- further dose reduction to 75%	10.7% (3/28), *n* = 2 in the high dose TT group
Intrathecal therapy	0% (0/28)

### Imaging findings on follow-up

#### Radiologic response

All cases (*n* = 28) could be reviewed at baseline and post-HDC, followed by *n* = 27 (m12), *n* = 24 (m24), *n* = 19 (m36), *n* = 18 (m48), *n* = 11 (m60) and *n* = 8 (>m60). Radiologic response following induction treatment revealed a CR(u) in 28.5% (*n* = 8), PR in 60.7% (*n* = 17), mixed response in 7.1% (*n* = 2), and PD in 3.6% (*n* = 1). After HDC/ASCT, CR(u) was observed in 75% (*n* = 21), and PR in 25% (*n* = 7), with no SD or PD (i.e., 100% radiological response rate).

#### WML burden and brain atrophy

**Fig. 2:**
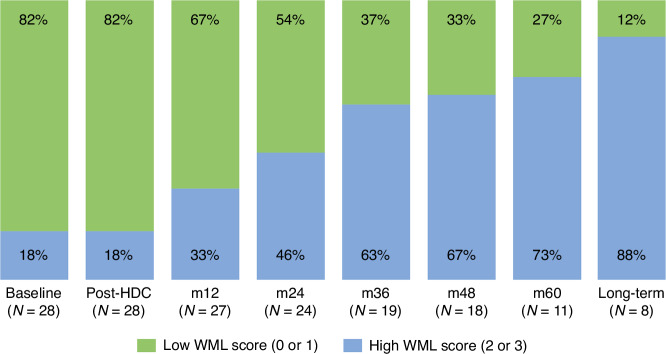
At baseline 17.8% (*n* = 5) showed a high WML burden with no immediate change post-HDC. Over time, the percentage of patients with high WML burden steadily increased: 33.3% (m12), 45.8% (m24), 63.1% (m36), 66.7 (m48), 72.8% (m60) and 87.5% (≥m60). In total, 17 of 28 patients presented with high WML burden at latest follow-up, corresponding to an overall rate of 60.7%.

Summary data of WML burden and all other collected parameters (mFS, DWM, PWM, MTA, and GCA) is shown in Table 2 and Supplementary table 1. We observed a significant increase in pathological changes over time for mFS, DWM, PWM (*p* < 0.05 for each parameter). At baseline, 17.9% (*n* = 5) already presented with a pathological mFS (≥2), indicating a high WML burden. The rate of high WML burden increased consistently, reaching a maximum of 60.7% over time when averaged across all patients (baseline vs. follow-up, *p* = 0.014). Thus, *n* = 17 can be assigned to a high WML burden group and *n* = 11 to a low WML burden group. Strikingly, long-term survivors with follow-up periods >60 m (*n* = 7) reached high WML burden rates of 87.5% (Fig. 2). A conversion from low to high WML burden predominantly occurs within the first two years after HDC/ASCT. Until month 24, the median mFS remained at 1, with an increase to ≥2 starting from month 36 (Supplementary Fig. 1). cMRIs beyond month 60 revealed highest median mFS with 2.5. A deterioration in mFS scores resulted primarily from an increase in PWM scores (pathological conversion in 60.7%, *n* = 17), while DWM conversions were noted only in 21.4% (*n* = 6). Furthermore, pathological levels in PWM were reached earlier (m12) than in DWM (m60, Table 2). The WML burden at latest follow-up correlated positively and significantly with the baseline mFS (*p* = 0.004).

**Table 2:** Neurocognitive and Quality of Life evaluation in follow-up. Mini-Mental State Examination (MMSE), Quality of Life (QoL).

	Baseline	post-HDC	m12	m24	m36	m48	m60	>60 m	Overall
N	28	28	27	24	19	18	11	8	28
mFS, median (SD)	1 (1,03)	1 (0,99)	1 (1,01)	1 (1,14)	2 (1,24)*	2 (1,13)*	2 (1,13)*	2.5 (0,74)*	2 (1.1)*
PWM, median (SD)	1 (0,81)	1 (0,86)	2 (0,84)*	2 (0,97)*	2 (0,97)*	2 (1,07)*	2 (0,94)*	2.5 (0,53)*	2 (1.0)*
DWM, median (SD)	0 (1,01)	0.5 (1,02)	1 (1,07)	1 (1,22)	1 (1,38)	1 (1,39)	1 (1,43)	2.5 (1,35)*	1 (1.2)
GCA, median (SD)	1 (0,62)	1 (0,67)	1 (0,87)	1 (0,88)	1 (0,88)	1.5 (0,85)	1 (1,00)	2 (0,75)*	1 (0.9)
MTA, median (SD)	1 (0,62)	1 (0,64)	1 (0,67)	1 (0,77)	1 (0,75)	1 (0,61)	1.5 (0,90)	2 (0,64)*	1 (0.9)
Low WML (mFS 0 or 1)	82.1% (*n* = 23)	82.1% (*n* = 23)	66.7% (*n* = 19)	54.2% (*n* = 17)	36.8% (*n* = 16)	33.3% (*n* = 16)	27.3% (*n* = 20)	12.5% (*n* = 21)	39.3% (*n* = 11)
High WML (mFS 2 or 3)	17.9% (*n* = 5)	17.9% (*n* = 5)	33.3% (*n* = 9)	45.8% (*n* = 11)	63.2% (*n* = 12)	66. 7% (*n* = 12)	72.7% (*n* = 8)	87.5% (*n* = 7)	60.7% (*n* = 17)
Pathological GCA (≥2)	7.1% (*n* = 2)	10.7% (*n* = 3)	25.9% (*n* = 7)	33.3% (*n* = 8)	36.8% (*n* = 7)	50% (*n* = 9)	45.5% (*n* = 5)	75% (*n* = 6)	46.4% (*n* = 13)
Pathological MTA (≥2)	7.1% (*n* = 2)	10.7% (*n* = 3)	18.5% (*n* = 5)	29.2% (*n* = 7)	38.9% (*n* = 7)	31.3% (*n* = 5)	41.7% (*n* = 5)	75% (*n* = 6)	39.3% (*n* = 11)

*Pathological scores.

Atrophy scores, like all parameters, worsened over time and showed a late conversion to pathological values (m60) similar to DWM. They did not correlate with mFS scores during follow-up (Supplementary Table 1). Both GCA and MTA did not affect QoL (*p* = 0.860) or MMSE (*p* = 0.124). However, pathological GCA scores were significantly associated with age (*p* = 0.037) and ECOG (*p* = 0.019). In patients with both high WML and atrophy burden (*n* = 12), age was again the only decisive factor (*p* = 0.034).

### Neurocognitive and Quality of Life evaluation

Results of ECOG, MMSE and QoL evaluation are presented in Table 3. The ECOG performance status improved from a median of 1 at baseline to 0 at latest follow-up, although this change did not reach statistical significance (Wilcoxon paired, *p* = 0.952). MMSE and PROMs were obtained in *n* = 23 and *n* = 21 patients, respectively, with an average MMSE of 27.6 points (range 15-30) and average global health status (GHS) in the EORTC QLQ of 68.63% (range 16.6–100). The five functional subscales of the EORTC QLQ-C30 yielded the following averaged results: physical 74.87%, role 59.83%, emotional 80.67%, cognitive 64.39%, social 47.73% (Table 3). Correlation analyses revealed a significant negative correlation between MMSE and ECOG at latest follow-up (*p* = 0.014), i.e., better ECOG correlated with better MMSE results. There was no significant correlation between GHS and either MMSE or ECOG. Regarding the treatment components, TT and MTX dosage were significantly correlated with baseline ECOG (*p* = 0.05, i.e. worse ECOG was associated with low-dose TT and MTX reduction), while no significant correlations were found for GHS and MMSE (both *p* = 0.611). The MTX dosage showed no significant correlation with any MMSE or GHS assessments. Considering the patients age, we divided the cohort into two age groups ( < 65 y and ≥65 y). Comparing these groups, we observed no significant effect on baseline or latest ECOG (*p* = 0.162 and *p* = 0.213, respectively), GHS (*p* = 1.00), or MMSE (*p* = 0.169). As expected, the CCI was significantly different between the age groups (*p* = 0.012), while the HCT-CI showed a trend towards significance (*p* = 0.076).

**Table 3:** Results of imaging analysis. White matter lesions (WML), months (m), high dose chemotherapy (HDC), standard deviation (SD), * = pathological scores.

Follow-up Data	ECOG	MMSE	QoL: Global health status	QoL: Functional subscales
N	24/28	23/28	21/28	21/28
median value	0 (range 0–3)	/	/	/
average value	/	27.6 points (range 15–30)	68.63% (range 16.6–100)	physical 74.87% role 59.83% emotional 80.67% cognitive 64.39% social 47.73%
WML burden influence (low vs. high)	no (*p* = 0.138)	no (*p* = 0.208)	no (*p* = 0.702)	*Supplementary Table* 2

### Associations of imaging findings, neurocognition, and QoL

The latest WML burden during follow-up did not significantly affect ECOG (*p* = 0.138), MMSE (*p* = 0.208), or GHS (*p* = 0.702). Similarly, atrophy scores did not affect neurocognition (*p* = 0.452) or QoL (*p* = 0.603). However, the low WML burden group performed slightly better on the MMSE (average 28.40 vs. 26.92 points). Similarly, high WML burden did not significantly impact physical, role, emotional, cognitive, or social functioning, though average scores in all subfunctions were higher in the low WML burden group (Supplementary Table 2). When comparing the age groups ( < 65 y vs. ≥65 y), GHS (67.84% vs. 70.21%) and emotional functioning (80.0% vs. 82.1%) were better in elderly patients, without reaching statistical significance. Intriguingly, cognitive functioning was comparable between both groups (64.29% vs. 64.28%).

We observed no differences in low vs. high WML development stratified for sex (*p* = 0.253), CCI (*p* = 0.054), MTX dose (*p* = 1.0), TT dose (*p* = 0.66) or response before (*p* = 0.752), and respectively after HDC/ASCT (*p* = 0.250). A multifactorial ANOVA for risk factors influencing the development of high WML burden at the latest follow-up, including TT/MTX dose, CCI, sex, baseline age, ECOG, and baseline WML burden, identified baseline WML burden (*p* = 0.049) and age (*p* = 0.002) as significant influencing parameters. Again, MMSE and GHS remained unaffected (*p* = 0.452 and *p* = 0.603, respectively). The results of the ANOVA analysis were supported by additional inferential statistics. When comparing the low vs. high WML burden groups, a significant difference in age (*p* = 0.003) was observed, with the high WML burden group being older. Correlation analysis showed significant results between age and both baseline WML burden (*p* = 0.002) and latest WML burden (*p* = 0.035). However, when comparing age groups (<65 years, *n* = 18 and ≥65 years, *n* = 10), we confirmed the significant influence of age on the latest WML burden (*p* = 0.041), but not on the baseline WML burden (*p* = 0.315).

**Fig. 3:**
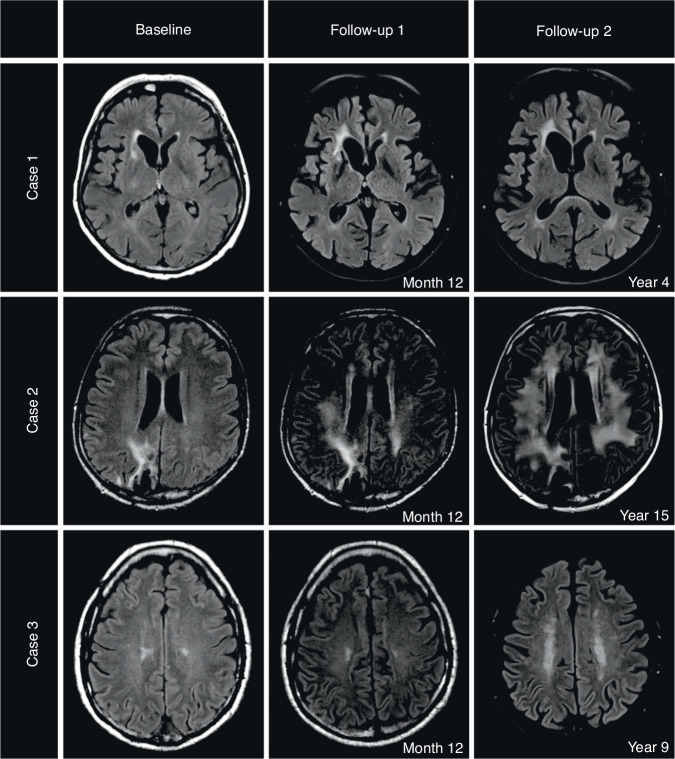
Exemplary Time Courses of 3 PCNSL cases. Case 1: Normal brain aging in a female PCNSL patient, 63 years at diagnosis with a 4-year follow-up period. Low WML burden at baseline (score 0) and at latest follow-up (score 1). Neurocognitive status at year 4: ECOG 0, MMSE 30 points, GHS unknown, QoL subscales all >80%. Case 2: Early WML changes in a male PCNSL patient, 43 years at diagnosis with a 15-year follow-up period. Low WML burden at baseline (score 0) with early conversion to high WML burden at month 12 (score 2). The high WML burden persisted until year 15 (score 3 from month 36 onwards). Neurocognitive status at year 15: ECOG 0, MMSE 28 points, GHS 50%. Case 3: Late WML changes in a male PCNSL patient, 60 years at diagnosis with a 9-year follow-up period. Low WML burden at baseline (score 1) with late conversion to high WML burden at month 60 (score 2). The high WML burden persisted unchanged until year 9 (score 2). Neurocognitive status at year 9: ECOG 0, MMSE 28 points, GHS 91.67.

We present three patients with exemplary time courses in Fig. 3.

## Discussion

We present data on neuroimaging changes following HDC/ASCT in PCNSL patients, highlighting their impact on neurocognitive outcomes and bridging a significant knowledge gap in this area. We assessed WML burden on cMRI in these patients over an average follow-up period of 4.7 years and detected significantly higher WML burden (60.7%) compared to baseline (17.9%). This observation supports the findings of previous neuroimaging studies [17, 32]. Similarly, no significant differences were observed with respect to basic neurocognitive and QoL evaluation when comparing low vs. high WML burden [7, 10]. However, MMSE and EORTC-QLQ30 functional subscales results were slightly better in the low WML burden group.

When examining the WML localization, the increase in WML burden primarily originated in the PWM, where lesions also appeared earlier. Our study did not replicate the previously observed correlation between PWM burden and cognitive decline, nor could we prove an association between PWM burden and GCA [20]. PWM burden was only associated with worse ECOG status. This contrasts with cohort studies of healthy adults, where WML burden was associated with cortical atrophy and cognitive dysfunction [33]. Conversely, in our study, a higher DWM burden suggested worse QoL and neurocognition, although these findings did not reach statistical significance and were not detected using mFS. Thus, the PWM/DWM division may introduce unnecessary subjectivity and could be omitted in favor of using mFS to evaluate WML burden in future studies [20]. Furthermore, we paid particular attention to the factor age in the context of WML and proved a robust age effect in multifactorial ANOVA as well as in t-tests split by age groups ( < 65 vs. ≥ 65 y). The age group effect was not seen for baseline WML burden, but rather for WML burden during follow-up, suggesting that age is likely not the only factor for WML presence. A more suitable explanation would be to allude to an accelerated brain aging theory, which is influenced by various factors, including cancer and treatment-related neurotoxicity [34, 35]. The high proportion of PWM changes corresponds to this theory, as they are driven by combinatorial processes [36]. Presuming that HDC/ASCT-related WML only damage the local WM while not inducing diffuse brain damage, the lack of significant cognitive decline in most PCNSL cohorts to date could be better explained. In line with this, cognitive functioning was equally good in both age groups and pathological GCA scores did not appear to be responsible for worse neurocognition or QoL. Consequently, WML effects seem to be differently mediated in healthy and cancer patients. However, the published research data remain inconclusive, with both data supporting our findings (9, 10) and present contradictory results [11, 37].

It is challenging to determine whether the significant increase in WML burden represents an entity-independent effect after HDC/ASCT or a specific PCNSL pattern. We could not include a comparative control cohort as imaging surveillance is not intended for other entities with HDC/ASCT. We hypothesize that the increase in long-term WML burden is an entity-independent effect of HDC/ASCT. This is supported by small studies in breast cancer, which also showed notable WM changes after HDC, associated with neurocognitive and QoL decline [38, 39].

This study is unique due to its relatively large number of patients with the same cancer type, its longitudinal design with pre- and post-treatment cMRIs, and sufficiently long follow-up periods incorporating patient-reported outcomes. Its limitations include the small cohort size due to the rarity of the disease and the high number of patients lost to follow-up, making it unrepresentative of all PCNSL survivors. Additionally, the lack of pre-treatment neurocognitive evaluations and comprehensive examination of all cognitive domains may lead to an underestimation of cognitive dysfunction [8]. To date, the roughly standardized neuroradiological description of WML following HDC/ASCT makes their evaluation in daily routine challenging for patients and clinicians. Further research is needed to understand the consequences and risk factors of these developments.

## Conclusion

A significant part of PNCSL long-term survivors develop a high WML burden after HDC/ASCT. We found no specific contribution of patient or therapy characteristics on the evolution of WML burden. Intriguingly, even a high WML burden does not seem to affect QoL, basic neurocognition or performance status negatively. We do observe at least a trend suggesting a greater impact of deep WML on neurocognition compared to periventricular WML. Presumably, HDC/ASCT-induced WML are an imaging feature with minimal clinical significance.

## Supplementary information

Legends of Supplementary materials

Correlation coefficients and significance levels between mFS, DWM, PWM, GCA and MTA.

The Progression of Modified Fazekas Scoring (mFS) for Each Patient

EORTC QLQ30 subscales. Comparing the low vs. high WML burden group.

## Data Availability

For original data, please contact sina.beer@med.uni-tuebingen.de.
