# Supplementary material for: Clinical relevance of brain MRI changes in primary central nervous system lymphoma after high-dose-chemotherapy and autologous stem cell transplantation
Source: Bone Marrow Transplant. 2024 Aug 9;59(11):1506–12. doi: 10.1038/s41409-024-02382-4 (PMC11530371; doi:10.1038/s41409-024-02382-4)
Supplement: Supplementary file 1 — Legends of Supplementary materials [file 41409_2024_2382_MOESM1_ESM.docx]

Supplementary materials

**Supplementary Figure 1:** Swimmer-plot with the course of modified Fazekas scoring (mFS) for each patient over the whole follow-up period. Y-axis shows each individual case. Follow-up times and data points for mFS changes are represented horizontally at X-axis. The mFS is color-coded as follows: 0 = green, 1 = yellow, 2 = turquoise and 3 = blue. Scores of 0 and 1 belong to the low burden group, scores of 2 and 3 belong to the high burden group.

**Supplementary Table 1:** Correlation coefficients and significance levels between mFS, DWM, PWM, GCA and MTA.

**Supplementary Table 2:** EORTC QLQ30 subscales. Comparing the low vs. high WML burden group.
