# Supplementary figures and images for: Clinical relevance of brain MRI changes in primary central nervous system lymphoma after high-dose-chemotherapy and autologous stem cell transplantation
Source: Bone Marrow Transplant. 2024 Aug 9;59(11):1506–12. doi: 10.1038/s41409-024-02382-4 (PMC11530371; doi:10.1038/s41409-024-02382-4)

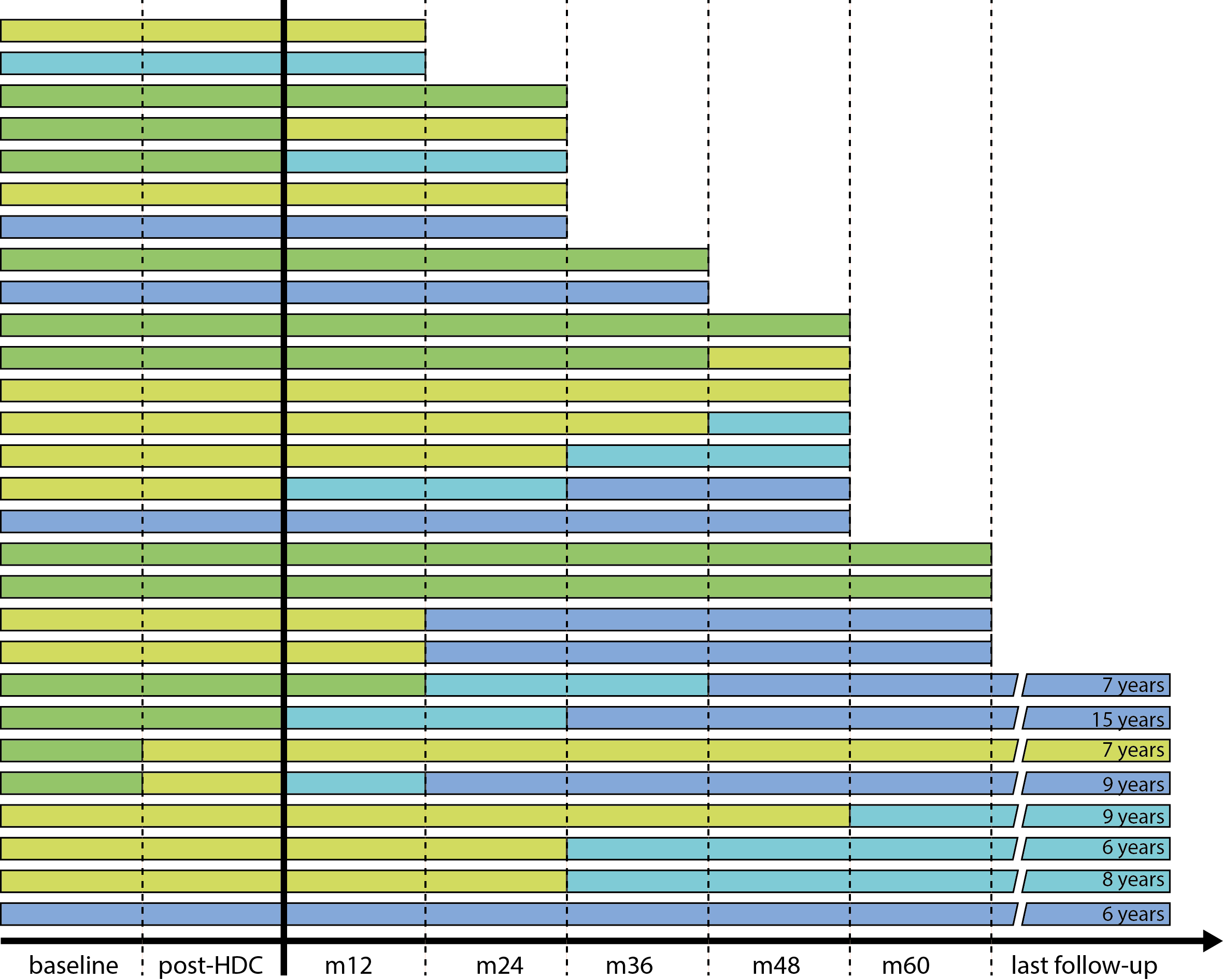

Supplement: Supplementary file 3 — The Progression of Modified Fazekas Scoring (mFS) for Each Patient [file 41409_2024_2382_MOESM3_ESM.png]
