# Supplementary material for: Clinical relevance of brain MRI changes in primary central nervous system lymphoma after high-dose-chemotherapy and autologous stem cell transplantation
Source: Bone Marrow Transplant. 2024 Aug 9;59(11):1506–12. doi: 10.1038/s41409-024-02382-4 (PMC11530371; doi:10.1038/s41409-024-02382-4)
Supplement: Supplementary file 3 [file 41409_2024_2382_MOESM2_ESM.docx]

		mFS	DWM	PWM	GCA	MTA
mFS	correlation analysis	1.000	0.951	0.956	0.465	0.188
	significance (2-sided)	.	**<0.001**	**<0.001**	0.246	0.655
DWM	correlation analysis	0.951	1.000	0.936	0.497	0.420
	significance (2-sided)	**<0.001**	.	**<0.001**	0.211	0.300
PWM	correlation analysis	0.956	0.936	1.000	0.354	0.189
	significance (2-sided)	**<0.001**	**<0.001**	.	0.390	0.654
GCA	correlation analysis	0.465	0.497	0.354	1.000	0.624
	significance (2-sided)	0.246	0.211	0.390	.	0.099
MTA	correlation analysis	0.188	0.420	0.189	0.624	1.000
	significance (2-sided)	0.655	0.300	0.654	0.099	.
