# Supplementary material for: Clinical relevance of brain MRI changes in primary central nervous system lymphoma after high-dose-chemotherapy and autologous stem cell transplantation
Source: Bone Marrow Transplant. 2024 Aug 9;59(11):1506–12. doi: 10.1038/s41409-024-02382-4 (PMC11530371; doi:10.1038/s41409-024-02382-4)
Supplement: Supplementary file 4 [file 41409_2024_2382_MOESM4_ESM.docx]

Functional subscales	low WML burden	high WML burden	p-value
Physical	0.786	0.717	0.558
Role	0.649	0.555	0.526
Emotional	0.875	0.749	0.295
Cognitive	0.722	0.583	0.28
Social	0.533	0.43	0.506
